# The Acceptability of Food Policies

**DOI:** 10.3390/nu13051483

**Published:** 2021-04-28

**Authors:** Romain Espinosa, Anis Nassar

**Affiliations:** 1 CNRS, CREM, Université de Rennes 1, 35065 Rennes, France; 2 Département d’Économie Politique, Université de Fribourg, 1700 Fribourg, Switzerland; anis.nassar@unifr.ch

**Keywords:** food policy, acceptability, survey

## Abstract

We propose and test a model of food policy acceptability. The model is structured in four levels: government, topic, policy, and individual. In this study, we focus on two levels that are actionable for policy-makers: the topic and policy levels. We assess nine factors using a first online survey with 600 UK nationals and replicate our results in a second survey with 588 participants. Our results suggest that three factors have a positive effect on acceptability at the topic level: awareness of the issue, the legitimacy of state intervention, and social norms. At the policy level, we report a positive effect of the policy’s expected effectiveness, its appropriate targeting of consumers, and the perceived support of the majority. On the other hand, more coercive interventions and those generating inequalities are judged to be less acceptable. Additionally, we report an interaction between awareness and coerciveness on acceptability. Participants who are aware of the issue were more likely to support coercive policies. We also find evidence for a trade-off between coerciveness, effectiveness, and acceptability, as more coercive measures are considered more effective, but less acceptable by participants. Our findings offer policy-makers, nutrition experts, and advocates for healthier and more sustainable diets a new and integrated understanding of the underlying factors that determine food policy acceptability.

## 1. Introduction

Population growth, combined with global changes in diets that are increasingly rich in sugars, refined fats, and animal-based products, is putting both our environment and public health under great stress. Food production is responsible for more than 25% of our greenhouse gas emissions [1], occupies close to half of all habitable land [2], and is the main driver of deforestation of tropical forests from the Amazon to South-East Asia [3,4]. Unhealthy diets are also responsible for a greater risk of morbidity and mortality than unsafe sex, alcohol, drug, and tobacco use combined [5]. On top of environmental and human health concerns, the expected increasing demand for animal-based proteins in the coming years is likely to consolidate intensive farming, which severely deteriorates animal welfare [6]. A dietary transition is thus one of humanity’s great challenges.

Recent works have shown that healthier and more sustainable diets can efficiently mitigate these issues, namely by reducing sugar consumption and shifting to plant-based proteins and unsaturated oils [5,7,8,9]. However, even though private action in this direction is producing positive results in some developed countries [10], decentralized and spontaneous dietary changes are likely to fall short in addressing this global challenge. Numerous consumers still underestimate the social impact of their diets [11], and a significant proportion of them refuse to acknowledge the consequences of their consumption [12,13]. Aware consumers might also be reluctant to change consumption habits that are part of their social identity and to which they are attached [14,15]. Even consumers who are effectively willing to change their diets might have difficulties following through, especially given that most food-related decisions are made unconsciously [16].

Appeals for public interventions supporting food transitions are therefore accumulating [17]. Authorities should, nonetheless, be cautious in their design and implementation, as policies regulating food that are not accepted can backfire [18,19]. Ensuring the acceptability of a public policy is therefore critical for its success. Widely accepted policies are also more likely to be implemented in the first place. Policies that benefit from a large popular support are indeed more likely to be enacted in direct democracies through referenda or in indirect democracies, in which political competition leads politicians to support popular interventions [20].

The objective of this paper is to offer a better understanding of the different factors that are actionable for policy-makers to increase the acceptability of policies regulating food. To that aim, we propose and test a level-based model of food policy acceptability. The governmental level includes macroscopic factors that influence the acceptability of *any* governmental intervention. The topic level adds factors that relate to the acceptability of public interventions *for a specific topic*. Citizens might have preferences for interventions depending on what the policy concerns (e.g., it is acceptable to regulate products containing cage eggs). The policy level corresponds to factors that influence the acceptability of a specific policy for a given topic (e.g., it is acceptable to have a GBP 0.1 tax on products that contain cage eggs). Finally, the individual level includes demographics and personal characteristics that may influence acceptability perceptions. In this study, we focus on the topic and policy levels. We restrict our attention to these two levels as we seek to understand what drives the variation of acceptability for different interventions across topics and policies for a given government and population.

In this work, we asked about 1200 UK nationals about their perceptions of different policies regulating a given food item (sugar, palm oil, or battery-cage eggs). The policies are composed of two education interventions (information campaigns, labeling) and four increasingly coercive market interventions (three levels of taxation and market withdrawal of the targeted product). Regarding the topic-level factors, we found that participants were more likely to support a food policy when they were more aware of the issue at stake, when they believed that it was legitimate to have collective rules regulating the product under scrutiny, and when there were strong social norms regarding the necessity to reduce the consumption of said product. As far as policy-level factors are concerned, we observed that policies were more popular when they were seen as more effective and when they targeted the appropriate group of consumers. On the contrary, consumers found coercive interventions and policies that generated inequalities less acceptable. Our results show the existence of a trade-off between coerciveness, effectiveness, and acceptability, as participants judged more coercive policies to be more effective, but also less acceptable. These conclusions can help guide policy-makers in the design of policies supporting a dietary transition.

## 2. A Model of Food Policy Acceptability

The interest in food policy acceptability has grown in the past decades, mainly following the increasing prevalence of obesity in developed countries [21]. The resulting literature has aimed at understanding the acceptability of food policies targeting obesity [22,23] and, more precisely, the support or aversion for taxation of sugar-sweetened beverages (see [24] for a review). The latest works on nutrition have shown a growing interest in the new dietary challenges faced by developed countries, such as the reduction of animal-based food consumption and the increase in plant-based protein intakes [5]. In addition, growing environmental and animal-welfare concerns further call for the enforcement of new food policies. This highlights the need for an integrated model of food policy acceptability that allows an understanding of the underlying factors that determine the acceptability of any food policy for any reason for intervention.

We propose a structuring of these factors in a multilevel model of food policy acceptability, which we relate to the existing literature, which often considers them in isolation [19,25,26]. In contrast, systems-like approaches that consider issues with a complex breadth of interconnected causes, interactions, and effects have gained relevance in recent years and have helped us to better understand critical food-related issues, such as obesity or unhealthy eating [27,28,29]. Scholars are also increasingly recognizing the importance of such comprehensive approaches for accurately assessing the (multiple) effects of a given policy and calling for their systematic use for the promotion and the evaluation of food policies [30,31,32,33]. Our work contributes to the development of such approaches by proposing a multilevel framework of food policy acceptability.

In our model, the governmental level includes factors that relate to perceptions about the government itself, such as the level of (mis-)trust in the government or the level of corruption, which can influence the acceptability of any policy proposed. At the topic level are factors that affect acceptability of an intervention due to a certain reason, such has limiting deforestation or preventing obesity. At the policy level are factors that determine acceptability of a specific type of policy. Finally, the individual level includes demographics, political leaning, and other personal characteristics. Although previous works showed that individual-level factors may influence acceptability, such as women finding interventions generally more acceptable [34,35], some evidence suggests that they have a limited influence compared to topic- and policy-level factors [22].

**Figure 1:**
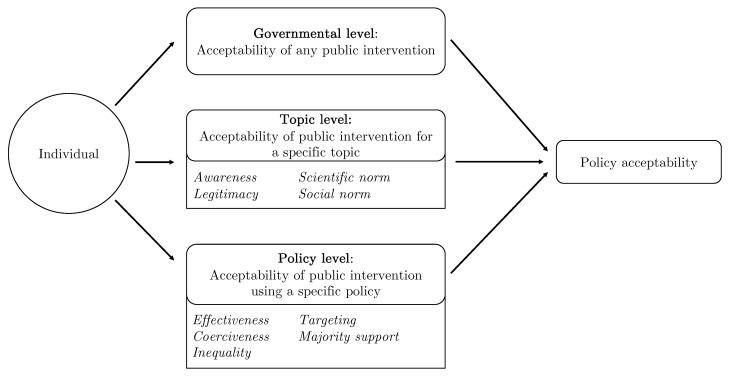
A model of food policy acceptability.

In this paper, we focus on the roles of the topic and policy levels in the acceptability of public interventions and how they vary across topics and types of policies for a given government and population. Our ultimate objective is to identify factors that are actionable by policy-makers to maximize the chances of a successful dietary transition. The model and the factors that were tested in the online survey are summarized in Figure 1 and Table 1.

### 2.1. Topic-Level Factors

The acceptability of a specific policy might depend on the underlying issue calling for governmental intervention, i.e., the topic of the policy. Previous research found that the level of awareness of the issue at stake is a key determinant of policy acceptability. Bos et al. [36] showed, in semi-structured interviews, that awareness of the problems of obesity leads to higher acceptability of different types of policies promoting healthier foods. These findings concur with the results of increasing acceptability linked to awareness of policies regarding other heath topics, such as smoking and drinking [34], or environmental policies, such as energy use [37].

Scientific research is regularly used as a basis for the implementation of new policies, but does it affect public acceptability? Policy-makers in the USA and New Zealand have suggested that more scientific evidence and/or a larger spread of scientific results could increase the public support for taxes on sugar-sweetened beverages [38,39], as there are still vast discrepancies between the established scientific consensus and public beliefs [24].

Social norms have also been established as a determining factor of the acceptability of public policies. Stok et al. [19] reported that an intervention aimed at increasing fruit intake is more accepted if participants are informed that trying to increase fruit intake is the norm in their peer group. A positive influence of perceived social norms was also reported in terms of the acceptability of transport pricing policies [40].

Last, views about the boundary between decisions that should remain inherently private and those that should be regulated by the state may influence the acceptability of public policies. As the body of evidence emphasizing the link between diets and chronic diseases has grown, governments have started taking action to influence diets [41]. In 2014, Mexico famously introduced a tax on sugar-sweetened beverages [42]. Since then, voices have risen to protest against what they consider to be privacy-invading policies, as illustrated in Washington, where citizens voted to prohibit new taxes on food grocery items in 2018 [43]. Several authors have also shown that, with respect to obesity, the ascription of responsibility for personal choices or environmental reasons influences the perception of the legitimacy of governmental intervention and, with it, the support for food policies [22,36,44]. The stronger the belief that the individual is responsible, the smaller the legitimacy of governmental intervention and the smaller the acceptability of public policies.

### 2.2. Policy-Level Factors

Despite being favorable to public intervention on a particular topic, citizens might consider a specific policy choice unacceptable. The perceived fairness of a policy has been consistently reported to increase its acceptability for both health interventions and environmental ones [26,45]. However, some researchers have questioned the relevance of explaining policy acceptability with fairness judgements, as the two notions of fairness and acceptability can be synonyms [46]. Consequently, we do not include fairness as a determinant of policy acceptability per se, but investigate two elements that determine opinions about the fairness of a policy: the types of individuals impacted by the policy and the inequalities expected to result from its implementation. It has indeed been established that the acceptability of nutrition policies depends on the extent to which certain groups are targeted, with higher acceptability for key groups, such as children and teenagers (for a review, see [47]). In addition, policies that are expected to worsen inequalities, i.e., those that will disproportionately impact citizens with low income, are expected to be less acceptable [36].

Moreover, citizens are also more prone to accepting interventions that are perceived as effective [26,36]. In a recent review, Reynolds et al. [25] reported that communicating the effectiveness of health-related policies is causal to increased public support. Similarly, the belief that a policy is supported by the majority improves its acceptability [48]. Participants are expected to be more willing to adapt their behaviors when they believe that a policy is effective and supported by their peers.

Restricting freedom of choice, i.e., the coerciveness of the policy, is, on the other hand, expected to negatively impact acceptability judgements. This effect has been documented for dietary interventions and for different health-related behaviors [22,34].

## 3. Survey

We investigated the role of the nine aforementioned factors in the determination of the acceptability of public interventions using an online survey.

### 3.1. Topics

We explored the determinants of policy acceptability for dietary interventions on three products that address the main challenges that food systems currently face: sugar (health), palm oil (environment), and cage eggs (animal welfare).

Sugar consumption has been shown to be a major driver of obesity and is associated with greater health risks [49,50]. This has led an increasing number of governments to undertake actions to limit sugar intake, such as sugary drink taxes (e.g., Australia, France, Portugal, Mexico, India). The production of palm oil significantly contributes to the increasing deforestation and represents a great threat to biodiversity and climate change [51]. These problems have been acknowledged by the EU, which discussed its partial ban for certain uses [52]. Cage eggs deliver the worst living conditions for egg-laying hens, with higher mortality and wound rates [53]. In 2020, 39% of the egg production still originated from cage-bound hens in the UK [54], while they are already banned in different countries.

### 3.2. Online Survey

We designed an online survey to elicit the factors that determine the acceptability of food policies. We adapted the survey to each of the three topics (between-subject design). The three questionnaires are displayed in the supplementary materials. Each questionnaire is made up of four parts.

First, we displayed six policies that could be implemented to regulate the consumption of snacks that contain the product targeted by the public policy. In the Sugar treatment, the public intervention focuses on snacks that have a high sugar content (more than 33 g of sugar per 100 g). In the Palm and Eggs treatments, the policies target snacks that contain palm oil or cage eggs, respectively. The six interventions are similar across topics: introducing a label to identify the targeted products, setting up an information campaign to educate consumers about the social impact of the products, introducing taxes of GBP 0.10, 0.30, or 0.50 for the targeted 30 g individual snacks, and removing the targeted snacks from the market (see Table 2).

Second, on a seven-point Likert scale, participants were required to indicate the extent to which they agreed with a list of statements regarding topic-level factors. The statements concerned the legitimacy of having collective rules on the consumption of the targeted product (*legitimacy*), the perception of the problems generated by the consumption of the targeted product (*awareness*), the social norms about whether the consumption of the targeted product should be reduced (*social norm*), and whether the product was over-consumed compared to the latest scientific recommendations (*scientific norm*).

Third, we asked participants about the degree to which they agreed with a second list of statements regarding policy-level factors for each of the six public interventions listed at the beginning of the survey (label, information campaign, GBP 0.10 tax, GBP 0.30 tax, GBP 0.50 tax, withdrawal from the market). Using a seven-point Likert scale, participants were required to report whether they agreed that the intervention was effective in reducing the consumption of the targeted product (*effectiveness*), targeted the appropriate group of consumers (*targeting*), was coercive (*coerciveness*), was acceptable for the participant (*acceptability*), if majority of citizens would agree with its implementation (*majority*), and if it would increase inequalities in society (*inequality*). As an attention check, the questionnaire displayed the *effectiveness* question twice (once in the first position and once in the second-lowest position).

Last, we asked participants whether they would vote “In favor” or “Against” the implementation (*vote*) of each of the proposed interventions individually (compared to the status quo, where nothing is done).

## 4. Results

### 4.1. Sample

We ran the survey on the online platform Prolific in February and early March 2020. Prolific is an online platform similar to Amazon MTurk, where people can subscribe to complete tasks for payment. Unlike Amazon MTurk, Prolific is mostly used for research purposes, and previous works concluded that Prolific yields better data than other online survey platforms (e.g., less dishonest participants, higher success rate in attention checks) [55].

To be eligible for the study, participants had to be born in the UK, to be UK nationals, and to have English as their native language. About 36,000 participants on the platform fulfilled our selection criteria (checked in early 2021) and were considered as active (i.e., in the last 90 days). All Prolific participants who fulfilled the above selection criteria could take part in our study until it reached the maximum number of participants. We defined an exclusion rule prior to the survey with Prolific by asking the *effectiveness* question twice and removing participants who gave significantly different answers to the same series of questions. We computed the average absolute deviation in answers given to the *effectiveness* questions for the six policies under consideration: $\frac{1}{6}\sum_{j=1}^{6}\mathrm{abs}\left({\mathrm{eff}}_{1j}-{\mathrm{eff}}_{2j}\right)$. Answers could take values between 1 and 7. We dropped participants whose average absolute deviations were greater than 2 (30 participants in Sugar, 32 in Palm Oil, and 24 in Eggs). We retained the answers to the first *effectiveness* question in the analysis.

In total, 600 participants completed the study and passed the attention check (200 respondents per topic). As it was not the focus of this study, we did not ask for demographics during the survey to avoid cross-contamination, but still exported the data previously gathered by Prolific. We were able to retrieve the demographics for 198 participants in Sugar, 191 participants in Palm, and 193 participants in Eggs (some participants revoked their consent to transmit the data to the researchers). We further asked Prolific for data on the BMI scores.

Descriptive statistics of the three samples are displayed in Table 3. We observed no statistical differences across topics regarding the sample composition: All Cohen’s d statistics were below 0.12, and none of the mean comparison tests (t-tests or proportion tests) rejected the null hypothesis. Participants were, on average, 35 years old (M = 34.67, SD = 11.45), mostly female (M = 0.73, SD = 0.44), and had a job (M = 0.72, SD = 0.45). One out of five respondents was a student (M = 0.20, SD = 0.40). The distribution of BMI scores was concentrated between 20 and 30 (51.92%). About one out of twenty participants was underweight (BMI < 20: 4.90%), and one out of five participants could be classified as obese (BMI > 30: 19.58%). In addition, one out of five participants refused to report or did not know their BMI (21.50%). Compared to the overall UK population, the sample was younger and more feminine and had a higher share of students.

### 4.2. Descriptive Statistics

**Figure 2:**
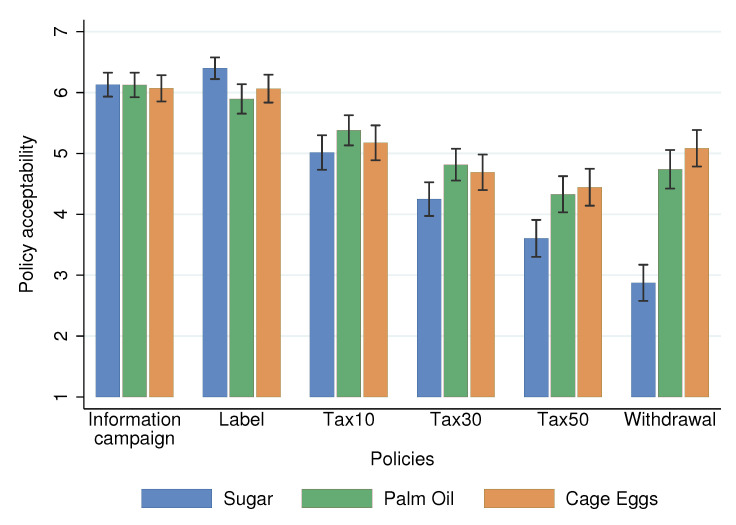
Reported policy acceptability: averages and 95% confidence intervals (spikes).

We begin by discussing the policy *acceptability* scores (Figure 2). First, we observed very high and similar levels of acceptability across the three topics for *labels* and *information campaigns*. Second, the level of *acceptability* decreased with the degree of *coerciveness*. We observed the highest *acceptability* scores for *labels* and *information campaigns*, followed by *tax10*. The lowest *acceptability* scores were for *withdraw* in Sugar and for *tax50* in Palm and Eggs (we address the differences between topics in the Discussion section). Third, interventions regulating palm oil and cage eggs displayed similar *acceptability* levels, but were more accepted than those related to sugar consumption (*tax10, tax30, tax50, withdraw*)

**Figure 3:**
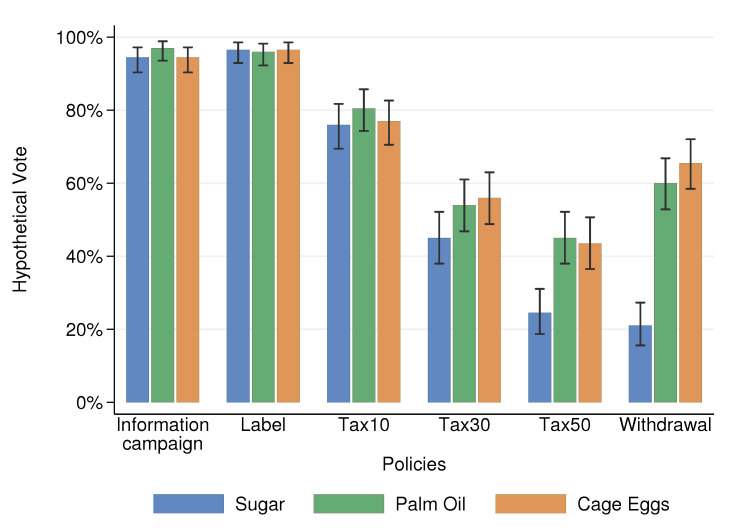
Hypothetical votes: averages and 95% confidence intervals (spikes).

The findings for the hypothetical votes (Figure 3) were very similar to those of *acceptability*, and *vote* was strongly correlated ($\hat{\rho }=0.65$, p<0.001, *N* = 3600, pooled data). The multivariate analysis below clusters the observations at the individual level to take into account the repeated observations in the data). We observed similar patterns, and some of the above figures are even more salient. The highest shares of votes were found in *label* and *information campaign*, followed by *tax10*. Here, the lowest share of votes was also for *withdrawal* for Sugar and for *tax50* for Palm and Eggs. Moreover, only 20% of the participants supported the withdrawal of the products with high sugar content compared to 60% for palm oil and cage eggs. Last, a majority of respondents rejected the highest level of taxation for palm oil and cage eggs, but they were more than 60% likely to accept the withdrawal from the market.

We now consider the topic-level factors, whose descriptive statistics are displayed in Table 4. First, we can see that for the three topics under scrutiny, at the aggregate level, participants tended to agree that (i) it is legitimate to intervene (*legitimacy*), (ii) there are negative effects associated with the targeted product (*awareness*), (iii) the product is over-consumed compared to scientific recommendations (*scientific norm*), and (iv) it is commonly accepted that consumption should be reduced (*social norm*). Indeed, all averages were greater than four points (the middle of the scale). Second, we can see that the intervention of the state was as legitimate for Palm as for Eggs (p=0.577), but it was significantly lower for Sugar (p<0.001). On the contrary, the perception of a *scientific norm* calling for a reduction in consumption was significantly greater for Sugar than for Palm Oil (p=0.001) and Eggs (p=0.002), and was similar for the two latter (p=0.939). Consumers displayed the largest *awareness* of negative effects for sugar consumption (M = 6.04, SD = 1.13), followed by palm oil (M = 5.44, SD = 1.33) and cage eggs (M = 4.58, SD = 1.54). Similarly, people were more likely to consider that the product was over-consumed for sugar (M = 6.37, SD = 0.98) than for palm oil (M = 5.58, SD = 1.23) and cage eggs (M = 5.28, SD = 1.28).

We analyzed the relationship between the topic-level factors and acceptability. To do so, we computed the average of the *acceptability* scores given to each of the six policies. We then correlated it with the topic-level factors (see Table A1 in Appendix A). We observed positive and statistically significant correlations between the four topic-level factors and acceptability. *Legitimacy* had the strongest correlation ($\hat{\rho }=0.449$). *Awareness*, *scientific norm*, and *social norm* also displayed positive and significant correlations close to $\hat{\rho }=0.21$.

Regarding the policy-level items, we observed significant differences across topics (see Table 5). Intervening against sugar consumption was perceived as less effective (p<0.001), less legitimate (p<0.001), and as having a lower social support (p<0.005) than interventions regulating palm oil and cage eggs. However, we did not find significant differences across topics in the average level of perceived coerciveness and the risks of generating inequalities. Regarding Palm and Eggs, we only found a weak statistical difference regarding the effectiveness of the policies (p=0.061): Regulating the consumption of cage eggs in snacks was expected to be more effective than for palm oil.

All policy-level items significantly correlated with policy acceptability (pooled correlation, p<0.001, N=3600). Public interventions were more likely to be accepted when a respondent believed that a majority of citizens would support it ($\hat{\rho }=0.576$), when they were perceived as effective ($\hat{\rho }=0.122$), and when they were thought to target the appropriate group of consumers ($\hat{\rho }=0.112$). On the contrary, policies were less likely to be accepted when they were seen as more coercive ($\hat{\rho }=-0.134$) and as generating more inequalities ($\hat{\rho }=-0.222$).

### 4.3. Multivariate Analysis

To identify the determinants of the acceptability of food policies, we estimated the following mixed linear model on *acceptability* and *vote*:

(1) $$\begin{array}{c} y_{ik}=\alpha_{0}+X_{i}\alpha_{1}+Z_{ik}\alpha_{2}+\mu_{i}+\phi_{k}+\epsilon_{ik} \end{array}$$

where $y_{ik}$ is either the *acceptability* or *vote* of individual *i* for policy *k*, $X_{i}$ is the vector of variables defined at the individual level (topic-level factors: *legitimacy, awareness, scientific norm*, and *social norm*) and demographics (gender, employment status, student status, age, BMI), and $Z_{ik}$ is the vector of variables defined at the policy–individual level (policy-level factors: *effectiveness, targeting, coerciveness, majority, inequality*). The terms $\mu_{i}$ are normally distributed individual effects, $\phi_{k}$ are (fixed) policy effects, and $\epsilon_{ik}$ are idiosyncratic error terms. The pooled regression further included dummy variables for the topics. The results presented below are robust to non-linear specifications (ordered probit and probit specifications, see Table A3 in Appendix A).

Estimates of the *acceptability* and *vote* are displayed in Table 6 and Table 7, respectively. We observed that *acceptability* and *vote* correlated with similar factors. The more one believes that it is legitimate to have collective rules for the topic under scrutiny (*legitimacy*), the more acceptable public interventions are. Policies that are expected to be more effective (*effectiveness*), to target the appropriate group of consumers (*targeting*), and to be accepted by the a majority of fellow citizens (*majority*) are also more likely to be accepted. On the contrary, coercive policies and those that are perceived as a source of inequalities in the society are less likely to be supported (*coerciveness* and *inequality*, respectively). On the other hand, the *scientific norm* did not significantly correlate with the level of *acceptability* in the multivariate analysis. Two variables showed weaker evidence. *Social norm* positively and significantly correlated with *acceptability*, but only partially with *vote* (significant for the pooled data and Eggs, but not for others). The second variable that showed weaker associations was *awareness*. It positively and significantly correlated with *vote* in all specifications, but not for *acceptability* (the association was significant only in Sugar), which we discuss in the next section.

Since we estimated a linear model in which all variables had the same range of values, we can compare the intensity of the contribution of each factor to the policy acceptability. The most influential factor is the expected majority support for a specific policy: An additional point in *majority* is associated with an increase of 0.5 points in *acceptability* and an increase of eight percentage points in *vote*. *Legitimacy* is the second-largest contributor to *acceptability* (+0.26) and *vote* (+4 pp). The policy’s effectiveness is estimated to be the third-largest contributor (*individual acceptability*: +0.17, *vote*: +3 pp). Perceived increases in inequalities and coercion have overall similar negative impacts on *individual acceptability* (−0.07) and *vote* (−1.5 and −1.3 pp respectively). The appropriate targeting of the policy has a similar but positive impact on *acceptability* (+0.12) and *vote* (+1.3 pp). Last, we ran a backward model selection analysis to understand which set of variables best explained *acceptability* and *vote*. To do so, we started with the full models (first column of Table 6 and Table 7) and removed the least significant variable. We then re-estimated our model with the new subset of variables and repeated the exclusion process. We stopped when all variables were significant at the 5% level. We computed the AIC and BIC scores at each step of the process. Control variables, individual random effects, and policy fixed effects were maintained in all specifications. The results suggest that a seven-component model fits the data best for *acceptability* compared to an eight-component model for *vote*. The variable *scientific norm* was excluded for the two dependent variables, and *awareness* was rejected for *acceptability*, but accepted for *vote*.

### 4.4. Replication: Confirmatory Analysis

In order to test the robustness of our results, we proceeded to a statistical replication (see [56]). To do so, we pre-registered the above findings (AEARCTR-0006429) and collected new data. We specified in the pre-registration that we would invite 220 individuals to participate in our study for each topic. We further committed to using the same recruitment platform (Prolific). We applied the same screening criteria and collected the data on 16 September 2020. We used the same exclusion rule as for the main analysis, which we also specified in the pre-registration protocol. The new sample of participants was statistically similar regarding age, student status, and job (see Table A6 in Appendix A). Participants in this second study were slightly less female (63% vs. 73%) and had slightly lower BMI scores (BMI < 30: 58.4% vs. 54.5%).

**Figure A1:**
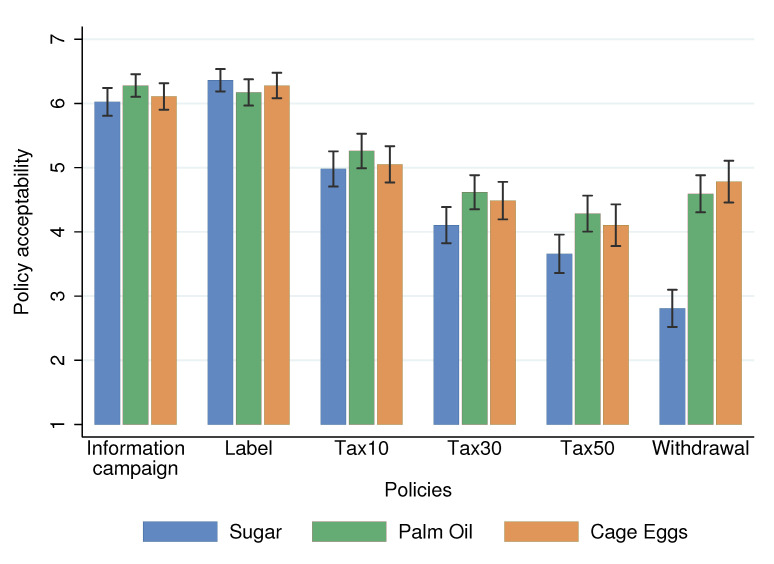
Reported policy acceptability—confirmatory analysis.

**Figure A2:**
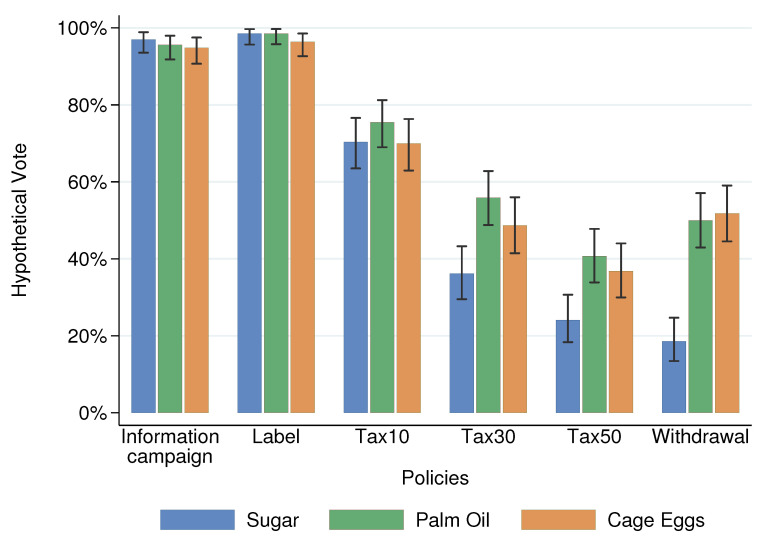
Hypothetical votes—confirmatory analysis.

We pre-registered that all variables but *scientific norm* were significantly associated with the acceptability of food policies. The results are displayed in Table 8 and show that we obtained similar results in the replication study (Table A7 and Table A8 in Appendix A show the full results). For the *acceptability* score, all variables but *scientific norm* were significant in the pooled analysis. Regarding *vote*, we obtained a similar pattern, except for the *social norm*, which was not significant. Finally, we can note that the average levels of *acceptability* showed similar patterns to those obtained previously. We reproduced Figure 2 and Figure 3 with the new data in Figure A1 and Figure A2 in Appendix A.2.

## 5. Discussion

### 5.1. Factors

All but one (scientific norm) of the nine factors tested correlate with acceptability judgments or hypothetical voting behavior. At the topic level, the perceived legitimacy of having collective rules to regulate the product at stake and favorable social norms are associated with stronger support for public intervention. At the narrower policy level, policies that are expected to have majority support, that target the appropriate group of consumers, that do not generate inequalities, and that are less coercive benefit from a larger acceptability. Our results concur with previous conclusions on food and health policy acceptability [22,23,34], as well as results from other fields [40,46,48]. Most importantly, they also validate the construct of these underlying factors having an effect at both the topic and policy levels and that this effect is consistent and valid for different topics of food interventions.

**Figure 4:**
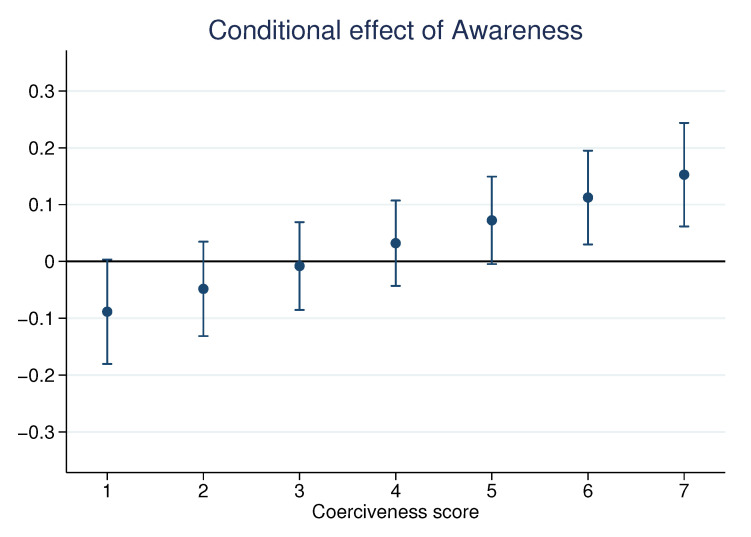
Conditional effect of *awareness* on policy *acceptability* in an interaction effect model. Note: Results of a linear regression of acceptability with policy fixed effects, topic fixed effects individual random effects, all identified acceptability factors, demographics, and an interaction term between awareness and coerciveness.

**Figure A3:**
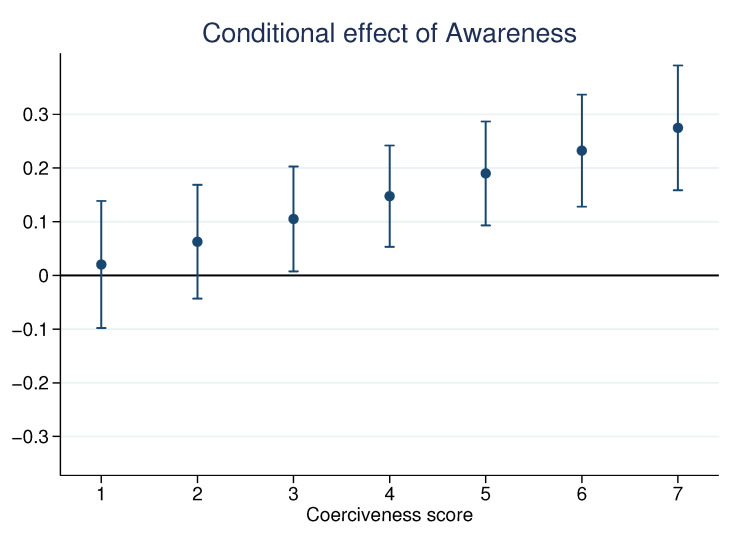
Conditional effect of *awareness* on policy *acceptability* in an interaction effect model—confirmatory analysis. Note: Results of a linear regression of acceptability with policy fixed effects, topic fixed effects, individual random effects, all identified acceptability factors, demographics, and an interaction term between awareness and coerciveness.

Surprisingly, in the first study, *awareness* did not significantly correlate with *acceptability* in the multivariate analysis. We explored the possibility that the effect of awareness on policy acceptability depends on the type of policy. To do so, we regressed the acceptability scores and hypothetical votes for each type of policy separately (Table A4 and Table A5 in the Appendix A). We observed that *awareness* is the only factor that displayed opposite and significant associations with *acceptability* and *vote*, depending on the policy. Aware consumers are more likely to support coercive measures, such as *tax50* or *withdrawal*, but they are also significantly less likely to support the implementation of *labels* or *information campaigns*. A possible explanation is that consumers who are aware of the problem do not consider labels coercive enough to address the problem and thus oppose their implementation. As a result, pooled regressions would lead to an average null effect of *awareness*, which would fail to take into account the underlying dynamic. To confirm this hypothesis, we investigated whether the impact of *awareness* depends on the coercion level. We ran a pooled regression similar to those presented in the previous section, but added an interaction term between *awareness* and *coerciveness*. The estimated relationship between *awareness* and *acceptability* is displayed in Figure 4 and confirms the aforementioned hypothesis: Awareness increases the acceptability of policies only if they are sufficiently coercive. This result was also pre-registered and successfully replicated (see Figure A3).

Our work also considered factors that affect the fairness of public interventions. Asking participants whether a policy is fair in their view is rather uninformative, as many of them might consider fair and acceptable as synonyms. To address this concern, we investigated two important dimensions that relate to fairness: whether the proposed policy targets the appropriate group of consumers and whether it generates inequalities in society. These two dimensions cover important aspects of fairness discussed by psychologists and economists, while ruling out the possibility of participants confounding the factors with the acceptability outcome [57]. Both inequality and appropriate targeting were reported to significantly correlate with acceptability judgements, confirming what other authors proposed [36,47], and supporting their relevance for future research.

Noticeably, *scientific norm* was the only factor for which we found no significant correlation with acceptability. Participants who agreed that consumption of the targeted product was higher than the recommendation of most recent scientific works did not consider policies to address the issue more acceptable. The benefits of using the scientific norm to support dietary changes therefore seemed limited. Importantly, we did not provide participants with informative scientific evidence, which might have yielded different results. Our results can only be interpreted as the use of scientific work as a form of authority. This result could be understood in the context of previous studies that have shown that simply telling people what to do is ineffective and can trigger reactance [19].

### 5.2. Policies

Our survey included six policies: labeling, information campaigns, taxing at low, intermediate, or high levels, and withdrawing the products from the market. Among these policies, we observed that labels and information campaigns benefit from a large acceptability: More than 90% of the participants would support the implementation of labels or information campaigns for the three topics under scrutiny. Policies that are not coercive and consist mainly of informing the population without changing the choice structure appear to be considered overwhelmingly acceptable, as reported by Mazzocchi et al. [22] for food and by Diepeveen et al. [34] for other health policies. Citizens are supportive of policies designed to give them the best tools to make informed decisions. On the contrary, the intermediate and high taxation levels showed significantly lower support, with the average level of acceptability decreasing with taxation intensity. It was equal to 5.19 for *tax10* versus 4.13 for *tax50* (*t*-test: p<0.001).

This trend corroborates the established trade-off that public authorities face between coerciveness and popular support. Coercive measures are appealing to policy-makers, as they are more likely to be effective, which participants also acknowledge. *Tax10* was, for example, perceived as less coercive than *tax50* (*t*-test: p<0.001), but also as less effective (*t*-test: p<0.001). Across policies and topics, we observed a strong correlation between reported coerciveness and reported effectiveness ($\hat{\rho }=0.427$, *p*< 0.001). Policy-makers must therefore choose the appropriate level of coercion that maximizes the change in behaviors while maintaining a sufficiently high level of acceptability for the population. In this paper, we did not investigate the actual capacity of a policy to change behaviors. However, it is worth noting that recent evidence indicates that certain information interventions, which are considered widely acceptable, can affect food consumption behaviors in the short and mid term [58]. When possible, it is also up to the policy-maker to consider nudges, which can, under certain circumstances, overcome the trade-off between coerciveness and effectiveness. Promising research has also already started in this direction [59,60].

### 5.3. Topics

We observed similar patterns in the *acceptability* and *vote* scores across topics. A notable exception was the change in acceptability between *tax50* and *withdrawal*. A priori, one could reasonably expect a lower acceptability for *withdrawal* than for *tax50*, as removing the possibility to buy the product should be judged as more coercive than taxing it. However, this decrease in acceptability was observed for the Sugar survey only, and people were more likely to accept the withdrawal than the highest level of taxation for Palm and Eggs. While participants indeed perceived the withdrawal as more coercive than taxation for all topics, we observed that their views diverged regarding its *effectiveness*: The effectiveness gain of shifting from high taxation to withdrawal was smaller for Sugar than for Palm and Eggs (Table A2). In addition, participants were more likely to think that a majority of citizens would support the withdrawal than for *tax50* in Palm and Eggs. Regarding sugar, we observed the opposite: Participants expected a larger social consensus for *tax50* than for the withdrawal. We observed a similar pattern in the confirmatory analysis.

These discrepancies can be explained by the fact that sugar differs from palm oil and cage eggs in terms of its impact. The negative consequences of consuming sugar are mostly borne by consumers themselves (increased risk of mortality or diseases), while they are mainly borne by others in the cases of palm oil (deforestation) and cage eggs (animals). Consuming sugar is thus more likely to be perceived as a personal choice, unlike palm oil and cage egg consumption, which can be seen as mainly a social issue. This idea is supported by the data, as we observed a lower legitimacy for interventions regarding sugar. In this case, coercive measures for sugar could be considered as a way to protect consumers from themselves, which can be perceived as paternalistic, while they would be considered as a way to protect others in the cases of cage eggs and palm oil.

### 5.4. Future Directions

Future research can build on this work in three main ways. First, our study focused on two levels of a four-level food policy acceptability model, as we excluded perceptions of the central government, judging them to be hardly actionable for policy-makers, and did not seek more individual information from our participants than what was already available. Future researchers would still benefit from incorporating these macro- and microscopic factors into their analyses. Cross-country comparisons could, for example, provide interesting insights on the variation of the effect of such factors in countries with different institutions and social capital.

Second, our research did not specifically measure the behavioral costs of policies. Policies with similar designs and pursuing the same objectives might still have very different behavioral implications. For example, citizens might have different acceptability judgements regarding the banning of meat compared to the banning of palm oil, even though both policies would help prevent deforestation. Other factors than those that we identified here might come into play. These could include citizens’ personal preferences, such as attachment to a certain part of their diet [15], or the ease of substitution with other products.

Third, our work reports correlations between factors and acceptability judgments, which does not allow us to rule out reverse causality. Motivated beliefs (see [61]) can, for example, lead a participant who dislikes a policy to judge it as ineffective. Similarly, the false consensus effect can influence perceptions of the majority’s opinion [62]. This would lead to a strong correlation between expected majority support and personal judgment of acceptability, which we observed. Importantly, a key element for the policy-maker is to determine the possibility of using the identified factors as tools to increase the acceptability of a policy. Future research should therefore intend to determine the causal relationships between factors and acceptability, as well as the most effective factors for influencing acceptability. Influencing topic-level factors might generate horizontal spillovers (i.e., increasing the acceptability for all types of policies for a given topic), while influencing policy-specific factors might generate vertical spillovers (i.e., increasing the acceptability of similar policies for different topics). Identifying spillovers could be of great interest for policy-makers and advocates of healthier and more sustainable diets to tailor their communication strategies and identify key leverages. Horizontal spillovers could be valuable for nutrition experts when they seek to improve the general acceptability of interventions for one specific topic with a possible range of policy options. Vertical spillovers would be useful, for instance, for policy-makers that seek to internalize externalities in food consumption with taxation across different topics.

### 5.5. Limitations

Future works could investigate the robustness of our results in terms of two dimensions. First, the participants in our study were not representative of the UK population. As discussed in Section 4.1, our participants were indeed younger and more feminine than the average UK population, and are more likely to be students. We cannot rule out the possibility that other types of participants would weigh the factors differently when they consider the acceptability of food policies. Second, the framework of our experiment is relatively general, as is the case with most experiments, and participants could view policies in a different way when they arise in a specific context. For instance, the support for the taxation of highly sweetened snacks could depend on other pre-existing policies. Replicating these surveys before and after the actual implementation of food policies could thus provide new perspectives.

## Figures and Tables

**Table 1:** Summary of factors.

Factor	Description
Awareness	The high consumption of (sugar | palm oil | cage eggs) causes serious problems for society.
Legitimacy	It is legitimate to have collective rules that govern the consumption of (sugar | palm oil | cage eggs).
Social norm	It is commonly accepted that (sugar | palm oil | cage eggs) consumption should be reduced.
Scientific norm	We consume more (sugar | palm oil | cage eggs) in our society than recommended by the (most recent scientific work | the most recent environmental scientific work | most recent scientific work on preserving animal welfare).
Effectiveness	The measure is effective in reducing the consumption of (sugar | palm oil | cage eggs).
Coerciveness	The measure is coercive.
Inequality	The measure will increase social inequalities.
Targeting	The measure will affect the appropriate group of consumers and producers.
Majority support	A majority of citizens would agree to implementing the measure.

**Table 2:** Summary of policies.

Policy	Description
Information campaign	Set up information campaigns to inform consumers about the impact of (sugar | palm oil | cage eggs) on (health | environment | animal welfare) and society.
Label	Display labels on snacks with (high sugar content | palm oil | cage eggs).
Tax10	Tax the snacks with (high sugar content | palm oil | cage eggs) by GBP 0.10 (for a 30 g individual snack, such as a cereal bar).
Tax30	Tax the snacks with (high sugar content | palm oil | cage eggs) by GBP 0.30 (for a 30 g individual snack such as a cereal bar).
Tax50	Tax the snacks with (high sugar content | palm oil | cage eggs) by GBP 0.50 (for a 30 g individual snack such as a cereal bar).
Withdrawal	Remove the snacks with (high sugar content | palm oil | cage eggs) from the market.

**Table 3:** Descriptive statistics for demographics.

	Descriptive Statistics				Effect Size (Cohen’s *d*) and Mean Comparison (*p*-Values)		
	All	Sugar	Palm	Eggs	Sugar = Palm	Sugar = Eggs	Palm = Eggs
Age	34.67	35.021	35.164	33.826	d=0.012	d=0.112	d=0.113
	(11.45)	(10.694)	(12.973)	(10.549)	p=0.906	p=0.272	p=0.271
Female	0.73	0.736	0.709	0.742	d=0.06	d=0.014	d=0.074
	(0.44)	(0.442)	(0.455)	(0.439)	p=0.559	p=0.887	p=0.47
Student	0.20	0.197	0.201	0.2	d=0.01	d=0.008	d=0.003
	(0.40)	(0.399)	(0.402)	(0.401)	p=0.919	p=0.939	p=0.979
Job	0.72	0.699	0.72	0.732	d=0.044	d=0.071	d=0.027
	(0.45)	(0.46)	(0.45)	(0.444)	p=0.665	p=0.486	p=0.793
BMI < 20	4.90%	3.63%	5.82%	5.26%			
20 ≥ BMI ≥ 24.9	26.92%	28.50%	23.28%	28.95%			
25 ≥ BMI ≥ 29.9	25.00%	23.83%	25.93%	25.26%			
30 ≥ BMI ≥ 34.9	11.36%	10.88%	13.23%	10.00%		χ${}^{2}$ = 11.26	
35 ≥ BMI ≥ 39.9	4.37%	5.18%	5.29%	2.63%		p=0.666	
40 ≥ BMI	3.85%	3.11%	2.12%	6.32%			
Refused to share	21.50%	22.28%	22.75%	19.47%			
BMI missing	2.10%	2.59%	1.59%	2.11%			
*N*	572	193	189	190			

Notes: (1) The figures here are the empirical means, with standard deviations in parentheses. (2) Absolute Cohen’s d values are reported. (3) Two-group mean comparison tests correspond to t-tests for Age and to proportion tests for Female, Student, and Job. (4) The total sample contains 600 participants. The figures show the descriptive statistics for the entire sample. Because of the missing values in some demographics, the final sample used for the regressions consists of 193 complete data for Sugar, 189 for Palm Oil, and 190 for Eggs.

**Table 4:** Descriptive statistics for topic-level factors.

	Descriptive Statistics			Effect Size (Cohen’s *d*) and Wilcoxon Rank-Sum Tests (*p*-Values)		
	Sugar	Palm	Eggs	Sugar = Palm	Sugar = Eggs	Palm = Eggs
Legitimacy	5.13	5.74	5.81	*d* = 0.397	*d* = 0.425	*d* = 0.056
	(1.73)	(1.25)	(1.43)	p= 0.001	p< 0.001	p= 0.193
Awareness	6.04	5.44	4.58	*d* = 0.486	*d* = 1.084	*d* = 0.600
	(1.13)	(1.33)	(1.54)	p< 0.001	p< 0.001	p< 0.001
Scientific norm	6.09	5.68	5.69	*d* = 0.342	*d* = 0.314	*d* = 0.008
	(1.17)	(1.23)	(1.37)	p< 0.001	p= 0.002	p= 0.573
Social norm	6.37	5.58	5.28	*d* = 0.71	*d* = 0.955	*d* = 0.239
	(0.98)	(1.23)	(1.28)	p< 0.001	p< 0.001	p= 0.019
N	200	200	200			

Notes: (1) The figures for the descriptive statistics are the empirical means, with standard deviations in parentheses. (2) Absolute Cohen’s d values are reported. (3) All respondents are considered here (i.e., 200 per treatment), including participants with missing demographics (which are excluded from the regression analyses below).

**Table 5:** Descriptive statistics for policy-level factors averaged over the six policies.

	Descriptive Statistics			Effect Size (Cohen’s *d*) and Wilcoxon Rank-Sum Tests (*p*-Values)		
	Sugar	Palm	Eggs	Sugar = Palm	Sugar = Eggs	Palm = Eggs
Effective	4.35	4.70	4.88	d= 0.366	d= 0.541	d= 0.188
	(0.98)	(0.93)	(0.98)	p< 0.001	p< 0.001	p= 0.037
Targeting	4.08	4.52	4.68	d= 0.421	d= 0.557	d= 0.142
	(1.07)	(1.06)	(1.08)	p< 0.001	p< 0.001	p= 0.119
Coercive	4.09	3.98	4.10	d= 0.098	d= 0.007	d= 0.102
	(1.11)	(1.26)	(1.20 )	p= 0.489	p= 0.835	p= 0.375
Majority	4.03	4.36	4.30	d= 0.35	d= 0.287	d= 0.049
	(0.90)	(0.98)	(1.04)	p= 0.001	p= 0.008	p= 0.591
Inequality	3.21	3.21	3.21	d< 0.001	d< 0.001	d< 0.001
	(1.25)	(1.23)	(1.34)	p= 0.93	p= 0.94	p= 0.994
N	200	200	200			

Notes: (1) The figures for the descriptive statistics are the empirical means, with standard deviations in parentheses. (2) Absolute Cohen’s d values are reported. (3) All respondents are considered here (i.e., 200 per treatment), including participants with missing demographics (which are excluded from the regression analyses below).

**Table 6:** Regression of policy acceptability scores.

	All	Sugar	Palm Oil	Eggs
Legitimacy	0.227 ***	0.174 ***	0.212 ***	0.300 ***
	(0.0329)	(0.0422)	(0.0738)	(0.0647)
Awareness	0.0338	0.167 **	0.0298	−0.0490
	(0.0382)	(0.0676)	(0.0701)	(0.0634)
Scientific norm	−0.00493	−0.0589	0.000393	0.0426
	(0.0369)	(0.0556)	(0.0693)	(0.0691)
Social norm	0.133 ***	0.0485	0.147 **	0.136 **
	(0.0392)	(0.0661)	(0.0729)	(0.0672)
Effective	0.169 ***	0.172 ***	0.137 ***	0.156 ***
	(0.0203)	(0.0314)	(0.0373)	(0.0359)
Targeting	0.122 ***	0.105 ***	0.114 ***	0.156 ***
	(0.0197)	(0.0309)	(0.0346)	(0.0355)
Coercive	−0.0704 ***	−0.111 ***	−0.0355	−0.0349
	(0.0168)	(0.0262)	(0.0286)	(0.0309)
Majority	0.498 ***	0.511 ***	0.467 ***	0.435 ***
	(0.0165)	(0.0277)	(0.0272)	(0.0308)
Inequalities	−0.0707 ***	−0.0252	−0.0961 ***	−0.111 ***
	(0.0159)	(0.0248)	(0.0272)	(0.0307)
Demographics	Yes	Yes	Yes	Yes
Individual RE	Yes	Yes	Yes	Yes
Policy FE	Yes	Yes	Yes	Yes
Topic FE	Yes	No	No	No
Number of individuals	572	193	189	190
Number of policies	6	6	6	6
Log-likelihood	−6243.19	−2034.42	−2012.94	−2104.98
Observations	3432	1158	1134	1140

Notes: (1) The figures here are the estimated coefficients, with standard errors in parentheses. (2) ** significant at 5%, *** significant at 1%. (3) Controls include: age, gender, student status, job, and body mass index.

**Table 7:** Regression of hypothetical votes in favor of the policies.

	All	Sugar	Palm Oil	Eggs
Legitimacy	0.0314 ***	0.0193 **	0.0266 *	0.0484 ***
	(0.00630)	(0.00863)	(0.0155)	(0.0106)
Awareness	0.0239 ***	0.0362 ***	0.0285 *	0.0174 *
	(0.00732)	(0.0138)	(0.0148)	(0.0104)
Scientific norm	0.000300	−0.00829	0.00793	−0.00503
	(0.00706)	(0.0114)	(0.0146)	(0.0113)
Social norm	0.0165 **	−0.00762	0.00874	0.0326 ***
	(0.00750)	(0.0135)	(0.0153)	(0.0110)
Effective	0.0301 ***	0.0282 ***	0.0281 ***	0.0311 ***
	(0.00484)	(0.00751)	(0.00922)	(0.00842)
Targeting	0.0132 ***	0.0169 **	0.0104	0.0140 *
	(0.00467)	(0.00734)	(0.00854)	(0.00816)
Coercive	−0.0129 ***	−0.0232 ***	−0.00579	−0.00387
	(0.00390)	(0.00616)	(0.00695)	(0.00688)
Majority	0.0785 ***	0.0773 ***	0.0703 ***	0.0752 ***
	(0.00389)	(0.00660)	(0.00670)	(0.00695)
Inequalities	−0.0152 ***	−0.00634	−0.0191 ***	−0.0224 ***
	(0.00368)	(0.00583)	(0.00665)	(0.00662)
Demographics	Yes	Yes	Yes	Yes
Individual RE	Yes	Yes	Yes	Yes
Policy FE	Yes	Yes	Yes	Yes
Topic FE	Yes	No	No	No
Number of individuals	572	193	189	190
Number of policies	6	6	6	6
Log-likelihood	−1292.22	−374.26	−420.45	−430.00
Observations	3432	1158	1134	1140

Notes: (1) The figures displayed are the estimated coefficients, with standard errors in brackets. (2) * significant at 10%, ** significant at 5%, *** significant at 1%. (3) Controls include: age, gender, student status, job, and body mass index.

**Table 8:** Results of the replication study: original and replicated estimates of the regressions of policy acceptability scores and hypothetical votes.

	Acceptability		Hypothetical Vote	
	Original	Replication	Original	Replication
Legitimacy	0.227 ***	0.170 ***	0.0314 ***	0.0177 **
	(0.0329)	(0.0423)	(0.00630)	(0.00797)
Awareness	0.0338	0.151 ***	0.0239 ***	0.0391 ***
	(0.0382)	(0.0482)	(0.00732)	(0.00906)
Scientific norm	−0.00493	0.0268	0.000300	0.0148 *
	(0.0369)	(0.0441)	(0.00706)	(0.00828)
Social norm	0.133 ***	0.134 ***	0.0165 **	0.0138
	(0.0392)	(0.0510)	(0.00750)	(0.00957)
Effective	0.169 ***	0.218 ***	0.0301 ***	0.0363 ***
	(0.0203)	(0.0188)	(0.00484)	(0.00466)
Targeting	0.122 ***	0.110 ***	0.0132 ***	0.0139 ***
	(0.0197)	(0.0178)	(0.00467)	(0.00438)
Coercive	−0.0704 ***	−0.101 ***	−0.0129 ***	−0.0185 ***
	(0.0168)	(0.0159)	(0.00390)	(0.00383)
Majority	0.498 ***	0.467 ***	0.0785 ***	0.0685 ***
	(0.0165)	(0.0176)	(0.00389)	(0.00429)
Inequalities	−0.0707 ***	−0.0488 ***	−0.0152 ***	−0.0136 ***
	(0.0159)	(0.0162)	(0.00368)	(0.00389)
Demographics	Yes	Yes	Yes	Yes
Individual RE	Yes	Yes	Yes	Yes
Policy FE	Yes	Yes	Yes	Yes
Topic FE	Yes	Yes	Yes	Yes
Number of individuals	572	588	572	588
Number of policies	6	6	6	6
Log-likelihood	−6243.19	−6357.29	−1292.22	−1418.71
Observations	3432	3528	3432	3528

Notes: (1) The figures here are the estimated coefficients, with standard errors in parentheses. (2) * significant at 10%, ** significant at 5%, *** significant at 1%. (3) Controls include: age, gender, student status, job, and body mass index.

## Data Availability

The scripts and data used to perform the analysis and generate this manuscript are available on GitHub (https://github.com/EspinosaRomain/FoodPoliciesAcceptability.git, accessed on 20 April 2021) and archived in Zenodo [63].

## Supplementary Materials

The following are available online at https://www.mdpi.com/article/10.3390/nu13051483/s1.

## Appendix A

### Appendix A.1. Supplementary Tables

**Table A1:** Correlation between topic-level factors and average acceptability.

	Acceptability (PCA)			
	Sugar	Palm	Eggs	All
Legitimacy	0.469	0.427	0.396	0.449
	[*p* < 0.001]	[*p* < 0.001]	[*p* < 0.001]	[p<0.001]
Awareness	0.389	0.326	0.235	0.207
	[*p* < 0.001]	[*p* < 0.001]	*p*< 0.001	[*p* < 0.001]
Scientific norm	0.127	0.312	0.308	0.220
	[*p* = 0.074]	[*p* < 0.001]	[*p* < 0.001]	[*p* < 0.001]
Social norm	0.299	0.396	0.228	0.210
	[*p* < 0.001]	[*p* < 0.001]	[*p* = 0.001]	[*p* < 0.001]

Notes: (1) The figures here are the estimated correlation coefficients, with *p*-values in brackets. (2) Acceptability obtained by taking the average of the acceptability scores over all policies.

**Table A2:** Summary statistics of *tax50* and *withdrawal*.

	Tax 50			Withdrawal		
	Sugar	PalmOil	Eggs	Sugar	PalmOil	Eggs
Acceptability	3.61	4.33	4.45	2.88	4.74	5.09
Vote	0.25	0.45	0.44	0.21	0.6	0.66
Effectiveness	4.99	5.34	5.39	5.36	6.06	6.32
Targeting	4.54	5.09	5.11	5.3	5.82	6.08
Coerciveness	4.83	4.59	4.79	5.32	5.05	5.31
Majority	2.37	3.05	2.8	2	3.13	3.34
Inequalities	4.76	4.55	4.35	3.18	3.18	3.71

Notes: The figures here are the empirical means.

**Table A3:** Robustness check: linear and non-linear specifications.

	Acceptability		Hypothetical Vote	
	Linear (Original)	Ordered Probit	Linear (Original)	Probit
Legitimacy	0.227 ***	0.225 ***	0.0314 ***	0.182 ***
	(0.0329)	(0.0342)	(0.00630)	(0.0367)
Awareness	0.0338	0.0410	0.0239 ***	0.136 ***
	(0.0382)	(0.0397)	(0.00732)	(0.0430)
Scientific norm	−0.00493	−0.0136	0.000300	0.000192
	(0.0369)	(0.0382)	(0.00706)	(0.0406)
Social norm	0.133 ***	0.170 ***	0.0165 **	0.0994 **
	(0.0392)	(0.0409)	(0.00750)	(0.0434)
Effective	0.169 ***	0.168 ***	0.0301 ***	0.198 ***
	(0.0203)	(0.0189)	(0.00484)	(0.0309)
Targeting	0.122 ***	0.113 ***	0.0132 ***	0.0885 ***
	(0.0197)	(0.0184)	(0.00467)	(0.0288)
Coercive	−0.0704 ***	−0.0824 ***	−0.0129 ***	−0.0861 ***
	(0.0168)	(0.0162)	(0.00390)	(0.0243)
Majority	0.498 ***	0.425 ***	0.0785 ***	0.385 ***
	(0.0165)	(0.0161)	(0.00389)	(0.0240)
Inequalities	−0.0707 ***	−0.0801 ***	−0.0152 ***	−0.0842 ***
	(0.0159)	(0.0145)	(0.00368)	(0.0206)
Demographics	Yes	Yes	Yes	Yes
Individual RE	Yes	Yes	Yes	Yes
Policy FE	Yes	Yes	Yes	Yes
Topic FE	Yes	Yes	Yes	Yes
Number of individuals	572	572	572	572
Number of policies	6	6	6	6
Log-likelihood	−6243.19	−4657.26	−1292.22	−1214.10
Observations	3432	3432	3432	3432

Notes: (1) The figures here are the estimated coefficients, with standard errors in parentheses. (2) ** significant at 5%, *** significant at 1%. (3) Controls include: age, gender, student status, job, and body mass index.

**Table A4:** Regression of policy acceptability scores by policy.

	Label	InfoCamp	Tax 10	Tax 30	Tax 50	Withdrawal
Legitimacy	0.0906 **	0.0821 **	0.369 ***	0.350 ***	0.387 ***	0.118 **
	(0.0420)	(0.0404)	(0.0555)	(0.0553)	(0.0581)	(0.0573)
Awareness	−0.120 **	−0.0247	−0.103	−0.00459	0.112 *	0.342***
	(0.0490)	(0.0470)	(0.0640)	(0.0636)	(0.0670)	(0.0659)
Scientific norm	−0.0412	0.0548	−0.0187	−0.0289	−0.0303	0.0757
	(0.0478)	(0.0456)	(0.0615)	(0.0614)	(0.0645)	(0.0637)
Social norm	0.0718	0.124 **	0.119 *	0.169 ***	0.164 **	0.171 **
	(0.0505)	(0.0486)	(0.0660)	(0.0651)	(0.0686)	(0.0682)
Effective	0.102 **	0.202 ***	0.187 ***	0.119 *	0.248 ***	0.195 ***
	(0.0410)	(0.0457)	(0.0545)	(0.0616)	(0.0565)	(0.0536)
Targeting	0.0944 **	0.00719	0.166 ***	0.176 ***	0.102 **	0.127 **
	(0.0389)	(0.0406)	(0.0541)	(0.0557)	(0.0498)	(0.0541)
Coercive	−0.0492	−0.0243	−0.0354	−0.0830 *	−0.0922 **	−0.0945 **
	(0.0333)	(0.0287)	(0.0477)	(0.0479)	(0.0460)	(0.0376)
Majority	0.474 ***	0.419 ***	0.330 ***	0.446 ***	0.456 ***	0.476 ***
	(0.0377)	(0.0365)	(0.0424)	(0.0440)	(0.0439)	(0.0413)
Inequalities	−0.107 ***	−0.0872 ***	−0.145 ***	−0.101 **	−0.168 ***	−0.0279
	(0.0379)	(0.0327)	(0.0435)	(0.0404)	(0.0384)	(0.0335)
Demographics	Yes	Yes	Yes	Yes	Yes	Yes
Topic FE	Yes	Yes	Yes	Yes	Yes	Yes
Observations	572	572	572	572	572	572
R${}^{2}$	0.383	0.350	0.318	0.359	0.406	0.521

Notes: (1) The figures here are the estimated coefficients, with standard errors in parentheses. (2) * significant at 10%, ** significant at 5%, *** significant at 1%. (3) Controls include: age, gender, student status, job, and body mass index.

**Table A5:** Regression of hypothetical votes by policy.

	Label	InfoCamp	Tax 10	Tax 30	Tax 50	Withdrawal
Legitimacy	0.00775	0.00558	0.0544 ***	0.0566 ***	0.0538 ***	0.0215
	(0.00586)	(0.00648)	(0.0124)	(0.0150)	(0.0141)	(0.0133)
Awareness	−0.0135 **	−0.0135 *	0.00814	0.0402 **	0.0445 ***	0.0672 ***
	(0.00684)	(0.00753)	(0.0143)	(0.0172)	(0.0162)	(0.0153)
Scientific norm	−0.00261	−0.00120	0.00898	−0.000777	−0.000688	0.00647
	(0.00667)	(0.00731)	(0.0138)	(0.0167)	(0.0156)	(0.0148)
Social norm	0.0115	0.0140 *	0.0248 *	0.0145	0.0253	0.0330 **
	(0.00704)	(0.00779)	(0.0148)	(0.0177)	(0.0166)	(0.0158)
Effective	0.0120 **	0.0189 **	0.0435 ***	0.0404 **	0.0369 ***	0.0255**
	(0.00572)	(0.00732)	(0.0122)	(0.0167)	(0.0137)	(0.0124)
Targeting	0.00122	0.00264	0.00937	0.0113	0.00736	0.0215 *
	(0.00542)	(0.00651)	(0.0121)	(0.0151)	(0.0121)	(0.0125)
Coercive	0.00543	0.00216	0.00566	−0.0145	−0.0241 **	−0.0274 ***
	(0.00464)	(0.00460)	(0.0107)	(0.0130)	(0.0111)	(0.00872)
Majority	0.0281 ***	0.0377 ***	0.0641 ***	0.102 ***	0.0927 ***	0.0878 ***
	(0.00526)	(0.00584)	(0.00952)	(0.0119)	(0.0106)	(0.00957)
Inequalities	−0.0118 **	0.00679	−0.0185 *	−0.0287 ***	−0.0322 ***	−0.0181 **
	(0.00529)	(0.00523)	(0.00976)	(0.0110)	(0.00931)	(0.00776)
Demographics	Yes	Yes	Yes	Yes	Yes	Yes
Topic FE	Yes	Yes	Yes	Yes	Yes	Yes
Observations	572	572	572	572	572	572
R${}^{2}$	0.138	0.151	0.239	0.254	0.296	0.415

Notes: (1) The figures here are the estimated coefficients, with standard errors in parentheses. (2) * significant at 10%, ** significant at 5%, *** significant at 1%. (3) Controls include: age, gender, student status, job, and body mass index.

**Table A6:** Sample comparisons on demographics.

	Descriptive Statistics		Effect Size (Cohen’s *d*)
			and Mean Comparison (*p*-Values)
	First study	Second study	First = Second
Age	34.67	35.81	d= 0.093
	(11.45)	(12.94)	p= 0.113
Female	0.73	0.63	d= 0.215
	(0.44)	(0.48)	p< 0.001
Student	0.20	0.20	d= 0.012
	(0.40)	(0.40)	p= 0.839
Job	0.72	0.64	d= 0.173
	(0.45)	(0.48)	p= 0.003
BMI < 20	4.90%	6.80%	
20 ≥ BMI ≥ 24.9	26.92%	31.63%	
25 ≥ BMI ≥29.9	25.00%	20.07%	
30 ≥ BMI ≥ 34.9	11.36%	8.50%	χ${}^{2}$ = 16.33
35 ≥ BMI ≥ 39.9	4.37%	3.91%	p= 0.022
40 ≥ BMI	3.85%	1.70%	
Don’t say	21.50%	25.34%	
BMI missing	2.10%	2.04%	
N	572	588	

Notes: (1) The figures for the descriptive statistics are the empirical means, with standard deviations in parentheses. (2) Absolute Cohen’s d values are reported. (3) Two-group mean comparison tests correspond to t-tests for age and to proportion tests for female, student, and job.

**Table A7:** Regression of *individual acceptability*—confirmatory analysis.

	All	Sugar	Palm Oil	Eggs
Legitimacy	0.170 ***	0.242 ***	−0.0114	0.155 *
	(0.0423)	(0.0539)	(0.0911)	(0.0895)
Awareness	0.151 ***	0.108	0.222 **	0.165 **
	(0.0482)	(0.102)	(0.0923)	(0.0754)
Scientific norm	0.0268	0.0239	0.0889	−0.00999
	(0.0441)	(0.0983)	(0.0719)	(0.0716)
Social norm	0.134 ***	−0.0328	0.219 ***	0.110
	(0.0510)	(0.116)	(0.0815)	(0.0817)
Effective	0.218 ***	0.186 ***	0.188 ***	0.234 ***
	(0.0188)	(0.0311)	(0.0318)	(0.0342)
Targeting	0.110 ***	0.126 ***	0.137 ***	0.109 ***
	(0.0178)	(0.0306)	(0.0296)	(0.0318)
Coercive	−0.101 ***	−0.107 ***	−0.0921 ***	−0.0620 **
	(0.0159)	(0.0280)	(0.0262)	(0.0280)
Majority	0.467 ***	0.556 ***	0.391 ***	0.414 ***
	(0.0176)	(0.0337)	(0.0295)	(0.0296)
Inequalities	−0.0488 ***	−0.0424	−0.0641 **	−0.0758 ***
	(0.0162)	(0.0283)	(0.0272)	(0.0289)
Demographics	Yes	Yes	Yes	Yes
Individual RE	Yes	Yes	Yes	Yes
Policy FE	Yes	Yes	Yes	Yes
Topic FE	Yes	No	No	No
Number of individuals	588	192	203	193
Number of policies	6	6	6	6
Log-likelihood	−6357.29	−2054.31	−2160.74	−2073.94
Observations	3528	1152	1218	1158

Notes: (1) The figures here are the estimated coefficients, with standard errors in parentheses. (2) * significant at 10%, ** significant at 5%, *** significant at 1%. (3) Controls include: age, gender, student status, job, and body mass index.

**Table A8:** Regression of *vote*—confirmatory analysis.

	All	Sugar	Palm Oil	Eggs
Legitimacy	0.0177 **	0.0351 ***	−0.00898	0.000990
	(0.00797)	(0.00959)	(0.0172)	(0.0182)
Awareness	0.0391 ***	0.0239	0.0627 ***	0.0337 **
	(0.00906)	(0.0181)	(0.0174)	(0.0153)
Scientific norm	0.0148 *	0.00535	0.0248 *	0.00985
	(0.00828)	(0.0173)	(0.0135)	(0.0145)
Social norm	0.0138	0.00386	0.00818	0.0217
	(0.00957)	(0.0205)	(0.0153)	(0.0166)
Effective	0.0363 ***	0.0300 ***	0.0246 ***	0.0474 ***
	(0.00466)	(0.00732)	(0.00836)	(0.00858)
Targeting	0.0139 ***	0.00850	0.0200 ***	0.0201 **
	(0.00438)	(0.00719)	(0.00767)	(0.00794)
Coercive	−0.0185 ***	−0.00918	−0.0168 **	−0.0236 ***
	(0.00383)	(0.00644)	(0.00663)	(0.00689)
Majority	0.0685 ***	0.0679 ***	0.0592 ***	0.0679 ***
	(0.00429)	(0.00784)	(0.00751)	(0.00730)
Inequalities	−0.0136 ***	−0.0187 ***	−0.0177 ***	−0.0105
	(0.00389)	(0.00648)	(0.00677)	(0.00705)
Demographics	Yes	Yes	Yes	Yes
Individual RE	Yes	Yes	Yes	Yes
Policy FE	Yes	Yes	Yes	Yes
Topic FE	Yes	No	No	No
Number of individuals	588	192	203	193
Number of policies	6	6	6	6
Log-likelihood	−1418.71	−393.14	−513.71	−463.37
Observations	3528	1152	1218	1158

Notes: (1) The figures displayed are the estimated coefficients, with standard errors in brackets. (2) * significant at 10%, ** significant at 5%, *** significant at 1%. (3) Controls include: age, gender, student status, job, and body mass index.
