# Supplementary material for: The Acceptability of Food Policies
Source: Nutrients. 2021 Apr 28;13(5):1483. doi: 10.3390/nu13051483 (PMC8145031; doi:10.3390/nu13051483)
Supplement: Supplementary file 1 [file nutrients-13-01483-s001.zip › nutrients-1134914-supplementary.pdf]

Supplementary Material
The acceptability of food policies

Romain Espinosa, Anis Nassar
March 15, 2021

Supplementary material: Questionnaire for Sugar

In this questionnaire, we are interested in the consumption of snacks that can be bought in supermarkets or in grocery stores. These snacks vary in different aspects, and in particular in the sugar content. We consider here that products have a "high sugar content" if sugar exceeds one-third of the product (i.e., more than 33g of sugar out of 100g of product).

We are interested in six measures that could be implemented to regulate the country's sugar intake through the consumption of snacks:

- Measure 1: display labels on snacks with high sugar content.
- Measure 2: tax the snacks with high sugar content by £0.10 (for a 30g individual snack such as a cereal bar).
- Measure 3: tax the snacks with high sugar content by £0.30 (for a 30g individual snack such as a cereal bar).
- Measure 4: tax the snacks with high sugar content by £0.50 (for a 30g individual snack such as a cereal bar).
- Measure 5: remove the snacks with high sugar content from the market.
- Measure 6: set up information campaigns to inform consumers about the impact of sugar on health and society.

You will face below a list of statements. You are asked to indicate to which extent you agree with each of the statements on a scale ranging from 1 (completely disagree) to 7 (completely agree).

- It is legitimate to have collective rules that govern the consumption of sugar.
- The high consumption of sugar causes serious problems for society.
- As a whole, it is commonly accepted that sugar consumption should be reduced.
- As a whole, we eat more sugar in our society than recommended by the most recent scientific work.

You will now face the second series of statements (in lines) which will apply this time to several measures designed to decrease the consumption of high sugar content products (in columns). You are asked to indicate to what extent you agree, between 1 and 7, with each of the statements for each of the measures.

Remember that you can choose from the following options:

- 1 - Totally disagree

- 2
- 3
- 4 - Indifferent
- 5
- 6
- 7 - Totally agree

	Label	£0.10 tax	£0.30 tax	£0.50 tax	Withdraw from the market	Information campaign
The measure is effective in reducing sugar consumption.	...	...	...	...	...	...
The measure will affect the appropriate group of consumers/producers.	...	...	...	...	...	...
The measure is coercive.	...	...	...	...	...	...
The measure is acceptable to me.	...	...	...	...	...	...
A majority of citizens would agree to implement the measure.	...	...	...	...	...	...
The measure is effective in reducing sugar consumption.	...	...	...	...	...	...
The measure will increase inequalities in society.	...	...	...	...	...	...

Now imagine that you have the opportunity to vote for each of the measures presented independently. You must indicate, for each of them, if you would support the implementation ("In favor"), or if you would prefer to do nothing ("Against").

Would you be in favor or against the implementation of the measure?

	In favor	Against
Label	○	○
£0.10 tax	○	○
£0.30 tax	○	○
£0.50 tax	○	○
Withdraw from the market	○	○
Information campaign	○	○

Supplementary material: Questionnaire for Palm

In this questionnaire, we are interested in the consumption of snacks that can be bought in supermarkets or in grocery stores. These snacks vary in different aspects, and in particular in the presence of palm oil in the ingredients. Some products contain palm oil while others don't.

We are interested in six measures that could be implemented to regulate the country's palm oil consumption through the consumption of snacks:

- Measure 1: display labels on snacks that contain palm oil.
- Measure 2: tax the snacks that contain palm oil by £0.10 (for a 30g individual snack such as a cereal bar).
- Measure 3: tax the snacks that contain palm oil by £0.30 (for a 30g individual snack such as a cereal bar).
- Measure 4: tax the snacks that contain palm oil by £0.50 (for a 30g individual snack such as a cereal bar).
- Measure 5: remove the snacks that contain palm oil from the market.
- Measure 6: set up information campaigns to inform consumers about the impact of palm oil on the environment and society.

You will face below a list of statements. You are asked to indicate to which extent you agree with each of the statements on a scale ranging from 1 (completely disagree) to 7 (completely agree).

- It is legitimate to have collective rules that govern the consumption of palm oil.
- The high consumption of palm oil causes serious problems for society.
- As a whole, it is commonly accepted that palm oil consumption should be reduced.
- As a whole, we consume more palm oil in our society than recommended by the most recent environmental scientific work.

You will now face the second series of statements (in lines) which will apply this time to several measures designed to decrease the consumption of high sugar content products (in columns). You are asked to indicate to what extent you agree, between 1 and 7, with each of the statements for each of the measures.

Remember that you can choose from the following options:

- 1 - Totally disagree
- 2
- 3
- 4 - Indifferent
- 5

- 6
- 7 - Totally agree

	Label	£0.10 tax	£0.30 tax	£0.50 tax	Withdraw from the market	Information campaign
The measure is effective in reducing palm oil consumption.	...	...	...	...	...	...
The measure will affect the appropriate group of consumers/producers.	...	...	...	...	...	...
The measure is coercive.	...	...	...	...	...	...
The measure is acceptable to me.	...	...	...	...	...	...
A majority of citizens would agree to implement the measure.	...	...	...	...	...	...
The measure is effective in reducing palm oil consumption.	...	...	...	...	...	...
The measure will increase inequalities in society.	...	...	...	...	...	...

Now imagine that you have the opportunity to vote for each of the measures presented independently. You must indicate, for each of them, if you would support the implementation ("In favor"), or if you would prefer to do nothing ("Against").

Would you be in favor or against the implementation of the measure?

	In favor	Against
Label	○	○
£0.10 tax	○	○
£0.30 tax	○	○
£0.50 tax	○	○
Withdraw from the market	○	○
Information campaign	○	○

Supplementary material: Questionnaire for Eggs

In this questionnaire, we are interested in the consumption of snacks that can be bought in supermarkets or in grocery stores. These snacks vary in different aspects, and in particular in the presence of cage-eggs in the ingredients. Some products contain eggs from laying hens kept in battery-cages while others don't.

We are interested in six measures that could be implemented to regulate the country's cage-eggs consumption through the consumption of snacks:

- Measure 1: display labels on snacks that contain cage-eggs.
- Measure 2: tax the snacks that contain cage-eggs by £0.10 (for a 30g individual snack such as a cereal bar).
- Measure 3: tax the snacks that contain cage-eggs by £0.30 (for a 30g individual snack such as a cereal bar).
- Measure 4: tax the snacks that contain cage-eggs by £0.50 (for a 30g individual snack such as a cereal bar).
- Measure 5: remove the snacks that contain cage-eggs from the market.
- Measure 6: set up information campaigns to inform consumers about the impact of cage-eggs on animal welfare and society.

You will face below a list of statements. You are asked to indicate to which extent you agree with each of the statements on a scale ranging from 1 (completely disagree) to 7 (completely agree).

- It is legitimate to have collective rules that govern the consumption of cage-eggs.
- The high consumption of cage-eggs causes serious problems for society.
- As a whole, it is commonly accepted that the consumption of cage-eggs should be reduced.
- As a whole, we eat more cage-eggs in our society than recommended by the most recent scientific work to preserve animal welfare.

You will now face the second series of statements (in lines) which will apply this time to several measures designed to decrease the consumption of high sugar content products (in columns). You are asked to indicate to what extent you agree, between 1 and 7, with each of the statements for each of the measures.

Remember that you can choose from the following options:

- 1 - Totally disagree
- 2
- 3
- 4 - Indifferent

- 5
- 6
- 7 - Totally agree

	Label	£0.10 tax	£0.30 tax	£0.50 tax	Withdraw from the market	Information campaign
The measure is effective in reducing the consumption of cage eggs.	...	...	...	...	...	...
The measure will affect the appropriate group of consumers/producers.	...	...	...	...	...	...
The measure is coercive.	...	...	...	...	...	...
The measure is acceptable to me.	...	...	...	...	...	...
A majority of citizens would agree to implement the measure.	...	...	...	...	...	...
The measure is effective in reducing the consumption of cage eggs.	...	...	...	...	...	...
The measure will increase inequalities in society.	...	...	...	...	...	...

Now imagine that you have the opportunity to vote for each of the measures presented independently. You must indicate, for each of them, if you would support the implementation ("In favor"), or if you would prefer to do nothing ("Against").

Would you be in favor or against the implementation of the measure?

	In favor	Against
Label	○	○
£0.10 tax	○	○
£0.30 tax	○	○
£0.50 tax	○	○
Withdraw from the market	○	○
Information campaign	○	○
